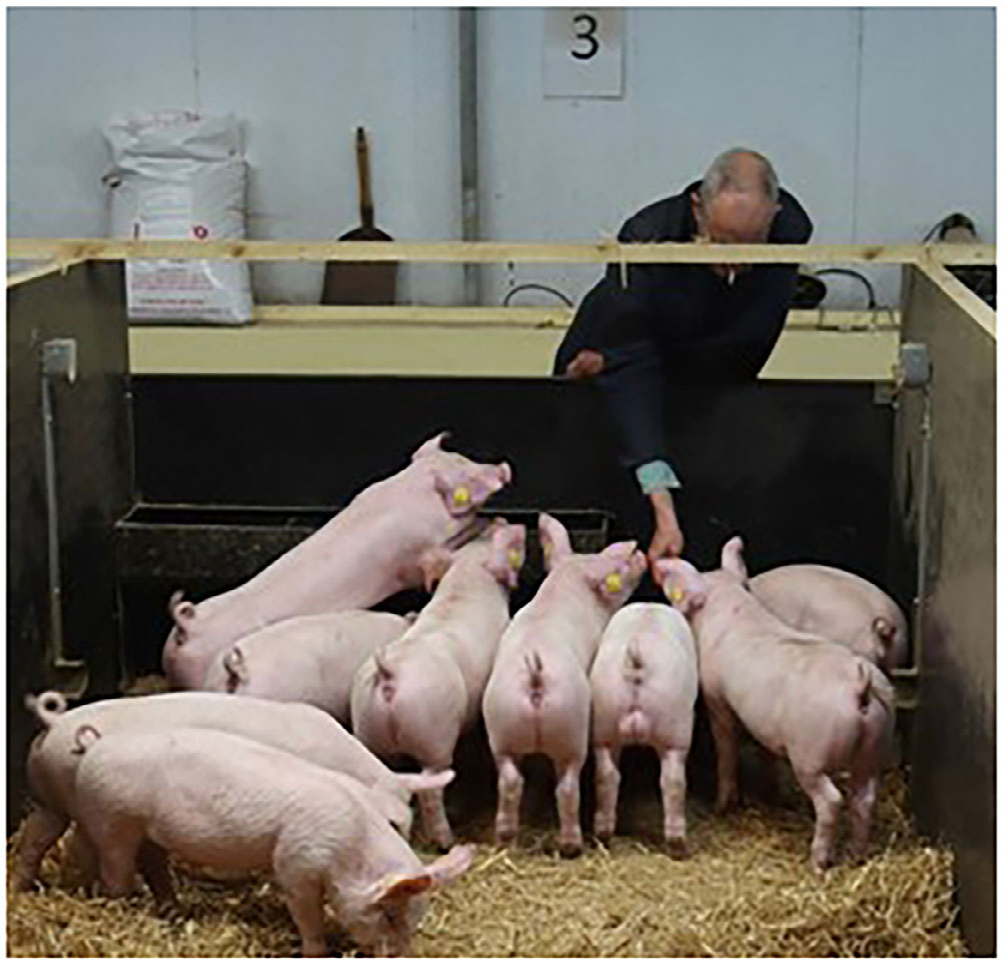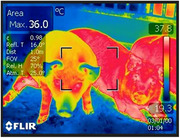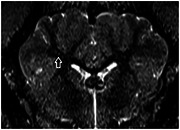# Transgenic Domestic Pigs for research in Vascular Contributions to Cognitive Impairment and Dementia

**DOI:** 10.1002/alz.085868

**Published:** 2025-01-03

**Authors:** Atticus H Hainsworth, Love Onwuzuruike, Misha Ramesh, Raimondo Ascione, Colin Berry, Leslie Bridges, Diana Cash, Eddie Clutton, Carola Daniel, Betty Gao, Jeremy D Isaacs, Simon Lillico, Jeremy Madigan, Daniel Meijles, James Nixon, Stephanie Osei, Adrian Ritchie, Monika Romanowska, Peter Tennant, Claire Warren, Joseph Westaby, C.Bruce A Whitelaw, Steven CR Williams

**Affiliations:** ^1^ St George’s University of London, London United Kingdom; ^2^ St George’s University Hospitals NHS Foundation Trust, London United Kingdom; ^3^ St Georges University of London, London United Kingdom; ^4^ University of Bristol, Bristol United Kingdom; ^5^ University of Glasgow, Glasgow United Kingdom; ^6^ Kings College London, London United Kingdom; ^7^ University of Edinburgh, Edinburgh United Kingdom; ^8^ St George's University Hospitals NHS Foundation Trust, London United Kingdom; ^9^ The University of Edinburgh, Edinburgh United Kingdom; ^10^ Institute of Psychiatry, Psychology and Neuroscience, King’s College London, London United Kingdom

## Abstract

**Background:**

Cardiovascular disease causes vascular dementia and contributes to most clinical dementia. This is embodied in the concept of vascular contributions to cognitive impairment and dementia (VCID). The potent endogenous peptide endothelin‐1 (ET1) causes small artery vasoconstriction and fibrosis. ET1 is implicated in microvascular disease and in VCID. There are few experimental animal models relevant to VCID [Hainsworth et al. 2017]. Pigs are higher mammals with a gyrencephalic brain and extensive subcortical white matter.

**Method:**

We engineered domestic pigs carrying additional copies of the ET1‐encoding gene *EDN1* under a Tet‐ON promotor, by lentiviral injection into blastocysts. We induced transgene expression for up to 8 days using oral doxycycline in young adults.

**Result:**

We studied ET1‐overexpressing (n = 6, 3F/3M, mean±SD age 184±61 days) and control animals (n = 5, 3F/2M, 149±41 days). Following doxycycline treatment we observed a wide range of transgene expression at mRNA level. Antibody labelling indicated a spectrum of ET1 abundance in brain and heart tissue. Heart weight was 460±106 g in ET1‐overexpressing pigs and 394±25 g in controls. Brain weight: 104±14.4 g in ET1‐overexpressing, 101±9.0 in controls. We will report phenotypes relevant to inflammation and blood vessel fibrosis.

**Conclusion:**

Adult‐onset *EDN1* induction in domestic pigs produces ET1 overexpression that is well‐tolerated to 8 days.

**Reference**: Hainsworth AH, et al. (2017) *BMC Med*.**15**(1):16. Translational models for vascular cognitive impairment: a review including larger species.